# Pumpkin and Pumpkin By-Products: A Comprehensive Overview of Phytochemicals, Extraction, Health Benefits, and Food Applications

**DOI:** 10.3390/foods13172694

**Published:** 2024-08-26

**Authors:** Roxana Nicoleta Gavril (Rațu), Florina Stoica, Florin Daniel Lipșa, Oana Emilia Constantin, Nicoleta Stănciuc, Iuliana Aprodu, Gabriela Râpeanu

**Affiliations:** 1 Department of Food Technologies, Faculty of Agriculture, “Ion Ionescu de la Brad” Iasi University of Life Sciences, 3 Mihail Sadoveanu Alley, 700489 Iasi, Romaniaflorin.lipsa@iuls.ro (F.D.L.); 2 Department of Food Science, Food Engineering, Biotechnology and Aquaculture, Faculty of Food Science and Engineering, Dunărea de Jos University of Galati, 800201 Galați, Romania; emilia.constantin@ugal.ro (O.E.C.); nicoleta.stanciuc@ugal.ro (N.S.); iuliana.aprodu@ugal.ro (I.A.); 3 Department of Pedotechnics, Faculty of Agriculture, Iasi University of Life Sciences, 3 Mihail Sadoveanu Alley, 700489 Iasi, Romania; florina.stoica@iuls.ro

**Keywords:** pumpkin, by-products, phytochemicals, food applications

## Abstract

A versatile and popular *Cucurbitaceous* vegetable, pumpkin has recently gained much attention because of its variety of phytochemicals and health advantages. Pumpkins are a type of winter squash, traditionally with large, spherical, orange fruits and a highly nutrient food. Pumpkin by-products comprise various parts, such as seeds, peels, and pulp residues, with their bioactive composition and many potential benefits poorly explored by the food industry. Pumpkin and their by-products contain a wide range of phytochemicals, including carotenoids, polyphenols, tocopherols, vitamins, minerals, and dietary fibers. These compounds in pumpkin by-products exhibit antioxidant, anticancer, anti-inflammatory, anti-diabetic, and antimicrobial properties and could reduce the risk of chronic diseases. This comprehensive review aims to provide a detailed overview of the phytochemicals found in pumpkin and its by-products, along with their extraction methods, health benefits, and diverse food and industrial applications. This information can offer valuable insights for food scientists seeking to reevaluate pumpkin’s potential as a functional ingredient. Reusing these by-products would support integrating a circular economy approach by boosting the market presence of valuable and sustainable products that improve health while lowering food waste.

## 1. Introduction

Pumpkins represent an extensive group of plants collectively known as pumpkins or squash in the family *Cucurbitaceae* and the genus *Cucurbita*. Cucumbers, melons, watermelons, pumpkins, squash, and gourds are the family’s valuable culinary plants. Their distinctive trait is that they are primarily herbaceous climbing vines with separate male and female blooms. In the tropics and warm temperate zones, the family is represented by more than 118 genera and 845 species. It belongs to the plant kingdom’s most morphologically diverse genera [1]. The pumpkin is thought to be the vegetable with the widest variety of traits, including size, shape, weight, and color. Below a central seed cavity, it contains a thick layer of edible pulp covered in a relatively hard rind. From July to September, pumpkins bloom, and from August to October, the seeds ripen [2]. Pumpkins are one of the greatest sources of bioactive compounds, and current studies on functional foods are increasingly focusing on them [3]. Pumpkin scientific classification refers to Kingdom—*Plantae*, Phylum—*Tracheophyta*, Class—*Magnoliopsida*, Order—*Cucurbitales*, Family—*Cucurbitaceae*, and Genus—*Cucurbita* L., and is classified as 75 species [4]. The pumpkin species have two distinct regions of origin: Mexico and Central and South America. Specifically, *Cucurbita moschata* and *Cucurbita pepo* are primarily associated with Mexico, while *Cucurbita ficifolia* and *Cucurbita mixta* are native to Central and South America [5]. However, *Cucurbita* is a small genus with about 27 species, the most cultivated and economically significant species being *C. moschata*, *C. maxima*, *C. mixta*, and *C. pepo*, which have been extensively investigated for their chemical makeup and biological activity. All around the world, there are numerous kinds and varieties of the cultivated edible fruit known as the pumpkin. Their fruit sizes are extremely diverse, ranging from several grams to hundreds of kilograms. One of the most varied domesticated species is the cultivated squash, *Cucurbita maxima*, which has at least five different varieties. The pulp, seeds, flowers, leaves, branches, and roots of the pumpkin plant are all utilized in daily diets around the world and in the pharmaceutical and cosmetics sectors [6].

**Figure 1:**
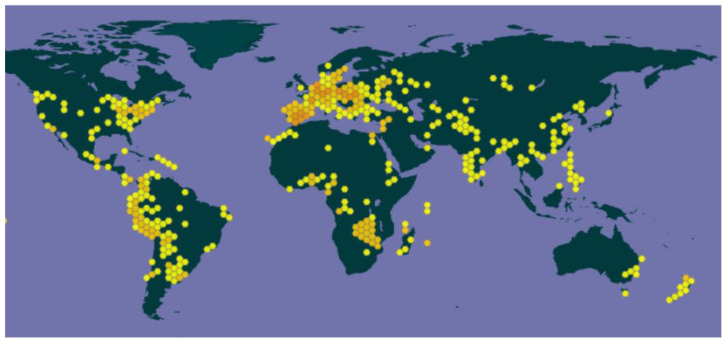
*Cucurbita maxima Duchesne* around the world [4].

Pumpkins are a significant cash crop that is grown extensively and consumed all over the globe (Figure 1). Pumpkins and squash are cultivated on a worldwide scale of around 3 million hectares, resulting in a total production of 27.832 million tons. China produces roughly 58% of the world’s pumpkins, followed by India with 20%, Ukraine with 4%, and Russia with 4%. Moreover, according to FAO [7], in 2020, Asia was the top global producer of pumpkins, squash, and gourds, with an output of 16,746,673 tons, accounting for 59.89% of the total. Europe follows with a production of 4,886,280 tons, representing 17.47%. The statistics related to the cultivation of pumpkins, squash, and gourds at the level of the member states of the European Union (E.U.-27) show that, in the last five years, the total production has registered an increase; therefore, in 2016, a production of 1,847,838 tons, and in 2020, the production reported by member states (EU-27) was 2,413,440 tons. Pumpkin by-products represent a common agro-food waste; data on the production of pumpkins, squash, and gourds in Romania indicate a value of 23,701 tons in 2016 and 26,850 tons in 2020, which is 1.03% lower compared to the previous year. Romanian edible varieties of *Cucurbita maxima*: “Alb mare” (from 2009), “Tudor” (from 2012), “De Bihor” (from 2006), “Mădăraș” (2010), etc. 

The pumpkin is one of the most underappreciated and underused food and medicinal plants. It is a remarkable vegetable that can be used both as a nourishing food and as medicine. Pumpkin (*Cucurbita maxima*) is an annual herbaceous plant cultivated for its fruit, flowers, and seeds. 

The nutritional content of pumpkins and other squashes includes sources of carbohydrates, fatty acids, fiber, carotenoids, vitamins, and minerals. The content of nutritional compounds depends a lot on the nature of the pumpkin varieties and the influence of environmental conditions (type of soil for planting, humidity, temperature, light, and mineral nutrients) [8]. Generally, the significant variability within a single species, based either on their subsequent variety or environmental factors and ripening stage, makes it difficult to establish clear qualitative or quantitative characteristics between the species. They can be consumed in a variety of ways. They serve the same purpose as animal feed. Fresh pumpkins can be consumed, cooked, baked, or processed into marinades, smoothies, soups, and juices. In the food industry, it is utilized as an ingredient in pastries, baked goods, biscuits, bread, candies, and baby food [9,10,11]. 

The formulation of pumpkin pulp is a common practice in the food sector, and it produces large amounts of waste. Wastes generated by certain industries have the potential to be utilized as raw materials for the production of various innovative products that offer notable health advantages and increased value to the industry. This approach allows for the reuse of industrial waste and reduces the need to depend on alternative sources for the manufacturing of different goods. The industry has been interested in the pumpkin wastes produced by industrialization, particularly the seeds, peel, and pomace, because of their beneficial constituents and high levels of carotenoids, dietary fiber, and polyphenols [12,13]. Thus, these leftovers possess significant potential as a reservoir of biomolecules with established health-enhancing properties for utilization in food items.

When pumpkins are processed, a substantial quantity of waste is generated in the form of (3.1–4.4%) and peels (2.6–16%), and the pulp constitutes 72–76% after processing. Pumpkin is commercialized worldwide, and its processing generates tons of seeds and peels as byproducts. The main sources of pumpkin waste are household waste, agricultural waste, and the food industry. The development of functional/nutritional foods, energy production, and biogas are only a few uses for pumpkin waste in the processing sectors. Pumpkin peels have been extensively used in the food industry as a natural colorant and dietary fiber source. 

Pumpkin seeds possess a significant amount of antioxidants and oil. The oil and protein content of pumpkin seed flour is around 51.0% and 36.5%, respectively [14,15]. 

Pumpkin seeds were shown to have a high protein content (48 ± 1%) when compared to other nutrients and other fruit seeds/kernels [16,17]. The research conducted by Łaba et al. [18] highlights the viability, efficiency, and cost-effectiveness of extracting and hydrolyzing pumpkin oilseed cake proteins using Bacillus subtilis. The technique not only improves protein recovery but also increases the value of agricultural waste. Pumpkin peels contain a lot of pectin [19] and Jun et al. [20] used them to extract an alcohol-insoluble polysaccharide and characterize the pectic polysaccharides they contained. 

As part of a sustainable approach, the current trend aims to combine the concepts of agriculture, industrial production, and circular economy by employing residues from the production of pumpkins as matrices to extract bioactive compounds of interest to the industry. Reducing, alternatively reusing, recycling, and recovering are used in place of the term “end-of-life”, which should result in a direct decrease in the amount of waste produced. Thus, it is anticipated that this approach will promote sustainable development, improve environmental quality, and increase economic prosperity [21].

Pumpkin seeds are used in salads, baked goods, and snacks due to their nutritional value and pleasant taste. Scientists are competently aware of the oxidizing and modulating effects of zinc on enzyme activation in the human organism during the COVID-19 pandemic, with pumpkin seeds being abundant in zinc. Pumpkin pulp and peels contain a significant number of phytochemicals, including β-carotene, total flavonoids, and total phenolics. Those compounds possess the capacity to increase the immune system and combat the effects of aging. Pumpkin peels are widely utilized in the food industry for their natural coloring properties and as a source of dietary fiber. The presence of terpenoids and polyphenols in this plant species makes it a valuable food addition. Carotenoids, of which β-carotene, α-carotene, neoxanthin, violaxanthin, and lutein stand out, are the most significant terpenoids in pumpkins [22]. The food sector is continuously altering as a result of an expanding population and increasing health concerns. The innovative approach of utilizing inexpensive and organic resources, such as agricultural and food byproducts, to produce innovative food products, has the potential to satisfy the increasing demand from consumers for environmentally friendly goods and transparent labeling. Given the difficulties faced by the food processing business, efforts are undertaken to optimize processing technology in order to reduce waste production. Wastes from the food processing sector are biologically treated and used as co-products, raw materials for other industries, or as food or feed. Pumpkin paste can be prepared in advance and stored in the freezer for future use, as inexpensive powders derived from pumpkin components can be utilized in the food and pharmaceutical sectors to effectively incorporate useful additives and nutraceuticals [23].

Pumpkin bioactive components are thought to protect against a wide range of illnesses, including coronary heart disease, cancer, diabetes, and hypertension. Pumpkin waste, generated during manufacture, processing, preparation, and distribution, can be transformed into sustainable products to prevent the loss of biomass and valuable nutrients, such as bioactive components [24]. These by-products offer significant nutritional benefits and present new market opportunities for the food, pharmaceutical, and cosmetic industries. Moreover, their reuse aligns with the United Nations’ sustainable development goals, reducing industrial waste and promoting sustainable growth through effective stabilization and extraction methods [25]. 

The present paper explores the basic composition of pumpkin waste, the necessary extraction techniques, and the applications of this waste in developing high-value goods within the framework of a circular bioeconomy. This review aims to highlight the most recent and crucial findings in the identification of pumpkin by-products as valuable sources of phytochemical constituents, the associated health benefits, the extraction methods of pumpkin by-products’ bioactive compounds, and the investigation of their potential diverse applications in various industrial sectors. Overall, this review makes a significant and beneficial contribution to sustainable pumpkin processing and circular economy.

## 2. Characterization of Pumpkin By-Products

**Figure 2:**
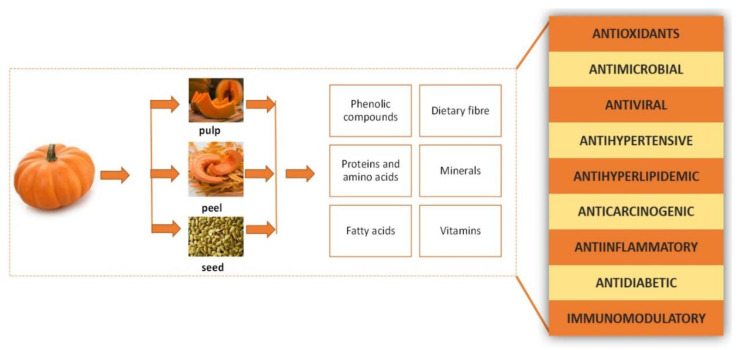
Biochemical components of pumpkin by-products and their role.

Because of the nutritional and health-protective properties of the proteins and oil from the seeds, as well as the polysaccharides from the fruit’s pulp, agriculture, food processing, the pharmaceutical industry, and the feed industry all have taken an increasing interest in pumpkin fruit and products derived from the pumpkin in recent years. Significant quantities of by-products in peels, seeds, and pomace are generated during the industrial processing of pumpkins into puree, juice, and pumpkin seed oil [26]. Figure 2 illustrates the types of pumpkin by-products and their corresponding biochemical elements. 

Pumpkin by-products are a valuable source of health-promoting compounds, including antioxidants, polyphenols, and carotenoids. Research indicates that diets abundant in antioxidants are associated with a reduced incidence of diabetes, cancer, and cardiovascular disease [2,8]. Additionally, studies have demonstrated that antioxidant compounds found in pumpkin seeds can help regulate blood sugar levels in animals with impaired glucose metabolism. Furthermore, the consumption of antioxidants has been linked to a lower risk of neurodegenerative diseases such as Alzheimer’s. Insufficient antioxidant levels in the body are also associated with increased oxidative stress, which has been implicated in the development of depression [5]. 

### 2.1. Pumpkin Pomace

The pomace produced as a by-product when pumpkins are used for food coloring agents is a heterogeneous matrix comprising various fractions of crushed skins, seeds, and mesocarp. This side stream, a low-value outlet for this by-product, is currently used as animal feed [27]. Although pumpkins are not typically used as a source of food fibers, several studies have examined the functionality of pumpkin polysaccharide fractions, concentrating on the characterization of cell wall-enriched powder from residual mesocarp [28] and pectic polysaccharides from peels and kernel cake [29]. The raw material displays a more complex chemical makeup and is also rich in proteins and lipids when made from industrially produced pumpkin pomace, which is derived from crushed seeds, skins, and mesocarp combined. In contrast to distinct isolated fractions in earlier studies, the inclusion of these distinct elements can offer different features and capabilities.

Pumpkin pomace is a good source of dietary fiber, vitamins, minerals, and carbs. The pumpkin pomace powder stands out due to its high carotenoid content of 35.55 mg/100 g dry weight (D.W.). In addition, it has high levels of minerals (calcium: 90 mg/100 g; phosphorus: 14 mg/100 g), cellulose (19.6%), hemicellulose (3.5%), and pectin (5.4%) [28]. 

Bakery and pastry goods can be fortified using pumpkin pomace powder. In the past, several scientists added pumpkin powder to various baked goods to examine the degree of suitability, modifications to the physico-chemical composition, impact on sensory parameters, and nutritional value of baked goods. When pumpkin peels and pulp were made into flour, their physicochemical examination showed that they are good sources of nutrients, and their use in making biscuits significantly impacted the biscuit industry [30]. 

### 2.2. Pumpkin Peels

Pumpkin peels are rich in bioactive components and include substantial quantities of fiber, protein, as well as minerals like calcium and magnesium. Nevertheless, the nutritional composition of pumpkin peels is lower in carbs, lipids, and potassium compared to pumpkin pulp [31]. Pumpkin peel contains a high concentration of carotenoids, which are pigment chemicals that can promote health [32]. 

Pumpkin peel includes the greatest amounts of total carotenoids (neoxanthin, β-carotene, violaxanthin, all-trans-lutein) and isomers (9-cis β-carotene, β-ionon, β-cyclocitral, 13-cis-β-carotene, α-ionon, dihydropseudoionon, tocopherol) among all fractions, but in different quantities in different varieties. According to Nuerbiya et al. [33], pumpkin peels include 10–40% of the overall carotenoid content present in pumpkins. Additionally, they serve as a valuable resource of provitamin A. Various factors, such as variations in the ripening stage, pedoclimatic circumstances, and harvest and postharvest treatment, can influence the content of carotenoids in pumpkin products and by-products [34]. Furthermore, the peels include fiber that is predominantly composed of cellulose [35], protein (9–17%), pectin, and amino acids [9]. Dietary fibers, such as pectin derived from pumpkin peels, have been shown to slow the digestion of starch, contributing to the management and prevention of diet-related conditions, including diabetes. Lutein, α-carotene, β-carotene, and zeaxanthin are some of the carotenoids found in pumpkin peels and have potential use in the food sector. Kurian and Kripan and [36] conducted a study to analyze the nutritional composition of pumpkin peel. They revealed that the peel contains 11.89 mg/100 g of β-carotene, 18.90 mg/100 g of ascorbic acid, 42.99 mg/100 g of iron, and 319.13 mg/100 g of phosphorus.

Badr et al. [37] determined the chemical makeup and biological value of pumpkin (*C. pepo*) peel powder [37]. The pumpkin peel powder contained 751.9 μg/100 g of β-carotene. Aspartic acid was discovered to be the most abundant amino acid in pumpkin peel powder (2.64%), followed by glutamic acid (2.53%) and leucine (1.2%). Comparatively, the peel section displayed the highest concentration of tryptophan compared to the pulp and seeds. The antibacterial properties of pumpkin peel extract were evaluated using the agar disc method. The results showed a 17 mm diameter of inhibition zone against the *Streptomyces viridochromogenes* and a 15 mm diameter of inhibition zone against the fungus *Mucor meihi*. These findings suggest that the extract has a wide range of antimicrobial activity.

Pectic polysaccharide is also present in significant amounts in pumpkin peels. Jun et al. [20] examined the chemical and physical properties of the pectic polysaccharides present in pumpkin skin. The researchers discovered that the pectic component derived from pumpkin peels has the ability to enhance the growth of gut bacteria and has implications for slowing down the absorption of glucose and bile acids. This suggests that pumpkin peels could be a valuable material for producing functional food.

### 2.3. Pumpkin Seeds

Although the edible portion of a pumpkin is its seeds, they are typically discarded as agro-industrial wastes. Pumpkin seeds are the most important component due to their high protein and low fat content. Flat, oval in shape, and pale green characterize the seeds. In addition to being utilized for both edible and medicinal uses, pumpkin seeds are utilized to produce oil and are eaten raw, boiled, and roasted in many countries. Pumpkin seeds are widely appreciated as a delicacy in several cultures and the seed oil has culinary and therapeutic uses due to its high concentration of bioactive compounds, including polyunsaturated fatty acids, essential amino acids, lutein, vitamins, phytosterols, γ-tocopherols, and β-carotene pigments, fibers, substantial amounts from micronutrients (P, Mg, Mn, K, and Ca) [38]. Karanja et al. [39] conducted research that discovered that pumpkin seeds contain high levels of crude protein, crude fiber, and crude oil. Pumpkin seeds exhibit a significant amount of polyunsaturated fatty acids, similar to the fatty acid composition observed in soybean and sunflower oils. According to a general examination of various pumpkin seed types, crude protein ranged from 14.05 to 33.29%, crude fiber from 11.69 to 24.85%, crude fat from 31.9 to 41.37%, and crude carbohydrates from 8.66 to 27.35%. The analysis of pumpkin seed extracts showed that they included substantial quantities of unsaturated fatty acids, particularly linoleic acid (26.18–81.21%), stearic acid (0.16–5.56%), oleic acid (15.56–30.79%), and palmitic acid (1.16–20.81%), which were the most abundant.

**Figure 3:**
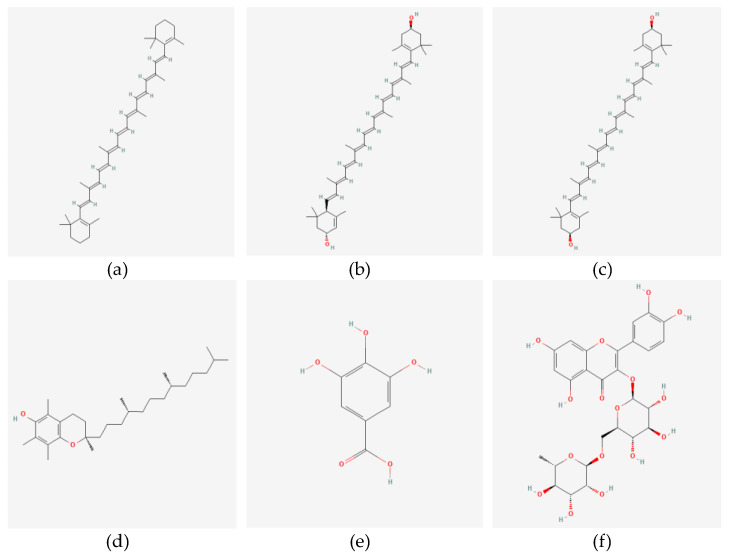
Examples of structures of various important bioactive compounds in pumpkin by-products: (**a**) β-carotene, (**b**) Zeaxanthin, (**c**) Lutein, (**d**) α-tocopherol, (**e**) Gallic acid, (**f**) Rutin [45].

The potential of pumpkin seed oil as both a nutraceutical and an edible oil has attracted considerable attention. Andjelkovic et al. [40] characterize it as a dichromatic, viscous substance with powerful antioxidant properties. Research has demonstrated that pumpkin seed oil exerts beneficial benefits on urinary issues, as well as on the regulation of bladder and urethral pressure, preventing hypertension, and reducing inflammation in the prostate [41,42,43]. Pumpkin seeds and pumpkin seed oil also contain significant amounts of vitamin E, an antioxidant. Pumpkin seed oil, with its fat content of 41.59% and protein content of 25.4%, is a highly abundant source of both protein and edible oil. Upon a thorough analysis of pumpkin seeds, it was determined that the moisture content was 5.2%, the crude fiber content was 5.34%, the total ash content was 2.49%, and the carbohydrate content was 25.19% [44]. Figure 3 illustrates the chemical compositions of various bioactive compounds present in pumpkin by-products.

## 3. Biochemical Components of By-Products from Pumpkins

### 3.1. Phenolic Compounds

Phenolic substances, a secondary plant metabolite that is prevalent in fruits and their by-products, have antioxidant, anti-mutagenic, and anti-inflammatory characteristics, among others [46]. According to Saavedra et al. [2], in the best operational conditions assessed, peels had a higher phenolic content than seeds, with values of 11 and 6.1 mg GAE/g D.W., respectively.

Pumpkin seeds have a complex phenolic profile that includes flavonoids, phenolic acids, and lignans. The primary chemicals identified were vanillic and caffeic acids, with values of 0.6 and 0.41 mg/100 g oil, respectively [47,48].

Upon examination of various pumpkin types, it was shown that *C. pepo* and *C. moschata* seeds contained higher levels of tocopherols than *C. maxima* seeds. In addition, the peels of pumpkins had a higher concentration of carotenoids than the seeds, especially in the case of *C. maxima.* β-carotene was found to be abundant throughout all types and varieties of pumpkins [9]. Ascorbic acid, chlorophyll a, and chlorophyll b were also determined by Blanco-Daz et al. [12], who obtained values for the first one ranging from 0.42 to 1.2 mg/g D.W. The same scientists demonstrated that peels had chlorophyll concentrations that were around 21 times higher than those of pulp.

### 3.2. Proteins and Amino Acids

Proteins are abundant in pumpkin by-products, notably in the seeds. According to Banerjee et al. [49], these bioactive chemicals have a range of biological properties, including antihypertensive, antioxidant, antibacterial, and immunomodulatory actions. Pumpkin seeds have been evaluated for protein content, with results ranging from 21 to 44% [14], and for the pumpkin peel, the values are lower, between 1.8 to 25% [50]. Kim et al. [9] measured the protein and amino acid contents of *C. maxima*, *C. pepo*, and *C. moschata* [9]. They found that *C. maxima* has a peel with a much greater protein content (17 g/kg fresh weight) than the other species (9.3 and 11 g/kg fresh weight, respectively). However, the protein content of *C. pepo* seeds was higher than that of *C. maxima* seeds (309 g/kg fresh weight vs. 275 g/kg, respectively). With the exception of aspartic acid, *C. maxima* peels had the highest levels of amino acids, but *C. pepo* seeds had the highest levels of amino acids due to their higher levels of arginine (64 mg/kg fresh weight).

### 3.3. Dietary Fiber

Pumpkin by-products can be viewed as a significant dietary fiber source that is used in the food sector. Fiber is a valuable tool for enhancing food products’ nutritional and functional qualities and/or physical characteristics, aiding in the prevention of illnesses including costiveness, cholesterolemia, cancer, and diabetes [35]. Due to the considerable amounts of cellulose and hemicelluloses present in these basic materials, pumpkin by-products (peels) have high levels of total dietary fiber (approximately 80%), with insoluble fiber constituting the majority of them [14].

Pectic oligosaccharides were extracted from the peel of pumpkins and separated into three groups (water, EDTA, and alkali-soluble oligosaccharides). The alkali-soluble fraction exhibited the highest levels of total sugar and uronic acid, measuring 25 and 46 g/100 g, respectively [20]. Pectin from pumpkin peel was extracted as efficiently as possible, and the resulting pectin and protein from pumpkin seeds were combined to generate biodegradable films. Pumpkin seeds and peels contain pectin, which can be extracted using acid hydrolysis. This pectin has great potential for replacing commercial pectin as a gelling agent and thickener in the food industry [51]. Compared to other pectin sources, pumpkin-derived pectin extracted from *Cucurbita maxima* with high molecular weight and a high degree of methoxylation offers advantages such as cytoprotective and antioxidant effects on cell models, and can be considered as an ingredient able to enhance the nutritional profile of the final food product [29].

### 3.4. Fatty Acids

The diversity of the cultivar, the degree of maturity, the growth conditions, and the preparation technique all influence the significant difference in the composition of fatty acids in pumpkin seeds. Thus, pumpkin seeds are valuable sources of lipids (rich in polyunsaturated fatty acids). They contain essential and non-essential fatty acids, which can prevent various conditions, such as cancer and other cardiovascular diseases. Pumpkin seeds have a fatty oil concentration of 45 to 60%. Pumpkin oil exhibits a substantial proportion of polyunsaturated fatty acids, comprising over 50% of the overall fatty acid content, alongside approximately 19.4% saturated fatty acids. The primary unsaturated fatty acids observed were linoleic acid, accounting for 21–55.6% of the total, followed by oleic acid, which accounted for 20.4–60.8%. The predominant saturated fatty acids were palmitic acid, ranging from 9.5% to 14.5%, and stearic acid, ranging from 3.1% to 7.7% [52]. 

A study conducted by Amin et al. [14] found that the lipid content of pumpkin seed oil varied between 18% and 55%. In Bangladesh, two kinds of pumpkin (*Cucurbita maxima*) (indigenous and hybrid) were examined for their FA profiles using GC/MS, and Amin et al. [14] found that the hybrid variety was particularly high in saturated fatty acids (capric, myristic, and stearic acids). Conversely, the native diversity of seeds exhibited a significant presence of unsaturated fatty acids, specifically oleic, linoleic, and linolenic acids. In their study, Nawirska-Olszańska et al. [48] observed that the oil content of *C. maxima* seeds is usually higher than comparable to *C. pepo* seeds. Oleic and linoleic acids were the primary fatty acids in *C. pepo*, *C. maxima*, and *C. moschata* [14]. 

In their investigation of *C. maxima* L. (var. *Berrettina*) pumpkin seed oil, Montesano et al. [53] found that the most prevalent fractions were polyunsaturated fatty acids and monounsaturated fatty acids (37.2% and 41.7%, respectively). The fatty acids that were most abundant were oleic and linoleic acids. Türkmen et al. [54] discovered that the unsaturated fatty acid content of edible pumpkin seeds collected from different locations in Turkey varied, up to 73.3%, with 18.4% oleic acid, 52.6% linoleic acid, and 1.27% linolenic acid being the main components; 27.7% of the fatty acids were saturated, with 16.4% being palmitic acid, and 11.1% being stearic acid.

### 3.5. Minerals

The maintenance of a healthy diet depends on the consumption of minerals. In addition to helping to cure and prevent hypertension, calcium is crucial for the growth of bones and teeth. Other micronutrients, like magnesium and potassium, play a crucial role in controlling blood pressure and regulating heartbeat [55]. The chemical composition of pumpkin (*C. pepo*) peel powder was determined by Badr et al. [37]. The calcium, iron, zinc, and copper values (mg/100 g dry weight) for pumpkin peel powder were 5571, 247.33, 42.92, and 12.91 mg/100 g, respectively. According to Idouraine et al. [56], squash seeds were shown to have significantly higher amounts of potassium (2069–2838 mg/100 g D.W.) compared to magnesium and calcium (which varied from 735 to 856 and 24–279 mg/100 g D.W., respectively). A vital mineral called zinc is in charge of improving the immune system and metabolism. Pumpkin seeds include peptides that are naturally associated with zinc, which are excellent minerals for promoting a healthy immune system [57,58].

### 3.6. Vitamins

Pumpkin has high levels of pro-vitamin A carotenoids, including lutein (a bright yellow color) and β-carotene (an orange hue). β-Carotene has a pro-vitamin A activity level of 100%. Once converted into vitamin A inside the body, it plays a crucial function in the immune system, night vision, development, and the formation and repair of epithelial tissue [59].

Pumpkin pulp contains 2–10 mg of vitamin C, 0.426 mg of vitamin A, and 9–10 mg of vitamin E per 100 g [14]. Vitamins like vitamin A, vitamin C, thiamine, folate, pyridoxine, riboflavin, niacin, and pantothenic acid were found in pumpkin seeds, in addition to, tocopherol or vitamin E [60,61]. δ-tocopherol, which made up about 42.2% of all the tocopherols in the Bejaoui variety’s seed oil, was the primary component, followed by α-tocopherol, γ-tocopherol [62].

## 4. Innovative Applications of Pumpkin By-Products

Around the world, pumpkins are grown for their use as food and medicine, and they are used to make various food products, including jams, syrups, jellies, purees, etc. In the last few decades, utilizing pumpkin wastes as functional food has presented new challenges. Snacks, bread, muffins, crackers, cookies, cakes, cereal bars, and other baked goods are all made with pumpkin seeds. The nutritional value of the manufactured product is improved by using pumpkin seed powder as a taste enhancer in dressings, soups, and baked goods [63,64,65].

Pumpkins have significant quantities of protein, fat, fiber, and tocopherol, all of which are abundant in pumpkin seeds. Additionally, they serve as an excellent reservoir of phytochemicals. Past studies have explored the use of fiber as a substance that can help prevent or treat diabetes. Additionally, all of these components possess nutritional qualities when consumed as part of a diet. Pumpkin, an abundant source of dietary fiber, is frequently added to culinary dishes to enhance their nutritional value and texture. Food engineers are using it more frequently to create unique foods with high nutritious value because its seed flour has a nutty flavor and an appealing green hue [66]. 

### 4.1. Natural Food Additives

**Figure 4:**
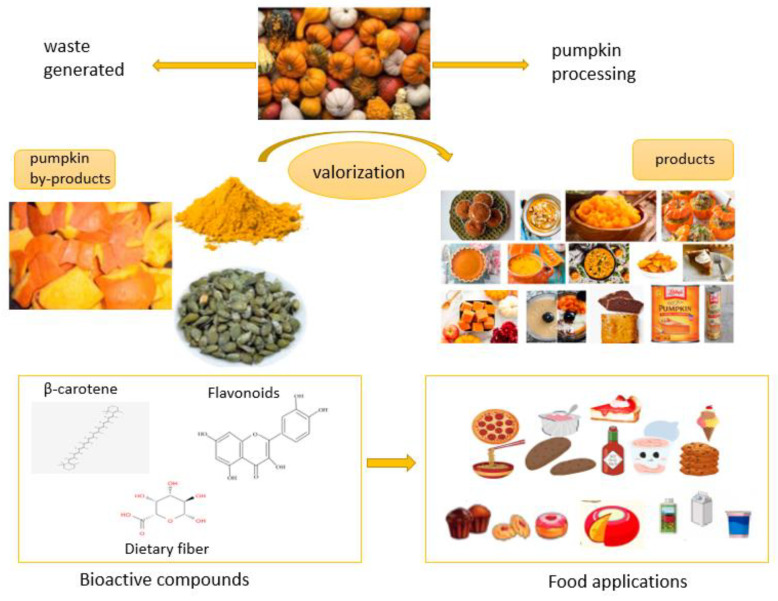
Valorization of pumpkin by-products and potential product applications.

Pumpkin by-products are more widely used in food processing due to their high nutritional content and potential health advantages. Pumpkin powder is an excellent source of dietary fiber, minerals, and antioxidants such as β-carotene. However, bread mixes and baked items that incorporate pumpkin contain a higher caloric content and are enriched with vitamin A. Figure 4 illustrates the utilization of pumpkin by-product powder in diverse food applications. Industrialists and food technologists are now pursuing the development of functional foods. 

Pumpkin flour, derived from both the seeds and peel of a pumpkin, can be utilized in a highly popular manner for utilizing pumpkin by-products. Apart from ascorbic acid and calcium, pumpkin peels are also rich in protein and fiber compared to the pulp. In the manufacture of bread, pumpkin peel flour was combined in part with wheat flour [11]. Bread made with 5% pumpkin peel flour has good qualities, including a high protein level, raw fiber, and a low carbohydrate content. By providing a brand-new category of nutritious and useful food, helping to decrease food waste, and creating a new added value for the utilization of discarded pumpkin peels, the usage of pumpkin peel flour stands out as a good market option. Burger Staichok et al. [11] used pumpkin hull flour to make bread as a technological application to partially replace wheat flour; 2.5% and 5% concentrations of pumpkin hull flour were used to make the bread. Although using pumpkin flour boosted the bread’s overall protein content, which is advantageous from a nutritional standpoint, there was a slight decline in cohesiveness, volume, and elasticity, which was to be expected given the low production of gluten.

Based on a nine-point hedonic sensory evaluation scale, Mishra and Sharma [67] made a pumpkin peel-based biscuit with 20% pumpkin peel flour that was the most appreciated. Based on its proximate evaluation (moisture, protein, fat, fiber, carbohydrate, energy, and ash value), the produced biscuit is rich in nutrients and can be utilized as a supplement in malnutrition and pregnant women. In order to make muffins, pumpkin powder was added to a wheat flour mixture. The nutritional, sensory, and physicochemical impacts of the addition were investigated [68]. When additional pumpkin powder was added to the baked muffins, the amount of β-carotene increased. Based on the sensory study, 20% pumpkin powder was shown to be the optimal proportion for acceptability; formulations above 20% had an adverse effect on the color and general acceptability of the product. In muffins, pumpkin flour was used in place of wheat flour to some extent, which resulted in a drop in saturated fatty acids and an increase in unsaturated fatty acids [69]. Children between the ages of 7 and 12 sensory-tested the product, and the results showed a 33% improvement in taste preference and general acceptability.

Many culinary products, including gluten-free noodles and pasta, have been made by partially substituting pumpkin flour (up to 50%) for corn flour. An analysis has been conducted on the sensory, physicochemical, and cooking yield aspects of the formulation products [70]. Pasta with 25% pumpkin flour had desirable qualities leading to improved color, texture properties, and sensory attributes of gluten-free pasta. The partial substitution of pumpkin flour with corn flour enhanced the moisture content, ash, cooking yield, and a* color parameter. Corn flour and vegetables processed with flour were used to make gluten-free spaghetti products [71]. The pumpkin combination tested highly in terms of its mechanical and sensory qualities, color, and general appeal.

The quality of the beef sausage that contained 30% dried pumpkin powder was enhanced [72]. Additionally, the formula was free of *Salmonella*. It enhanced protein and fat content while decreasing moisture content. Also, increasing the amount of dried pumpkin powder reduced pH and improved water holding capacity. Table 1 displays the industrial food applications for pumpkin by-products.

All ages and diverse cultures enjoy a variety of ground beef dishes, such as meatballs and burgers. According to a previous study, substituting flour and dry pumpkin seeds for red meat in beef patties resulted in a patty with a poorer texture and more water retention. [73]. To manufacture beef balls, powder is used in place of fat. Although the hamburger’s great qualities were allegedly lost, the cooking properties allegedly persisted. Moreover, opting for nutritious fat-free vegetable substitutes like soybean oil and pumpkin seed meal is a feasible choice [82]. In order to create a beef pellet with enhanced nutritional value and increased benefits, pumpkin seed meal was employed as a replacement for beef fat during the entire production procedure. Its nutritional value is assessed by its fatty acid content and health index, while consumer satisfaction is gauged by sensory evaluation. According to a study that looked at the impacts of including pumpkin seeds in chicken patties, these components might enhance the end product’s baking and lipid oxidation capabilities [73]. 

A study was conducted to examine the possibility of creating a novel ice cream recipe with a significant percentage of pumpkin pulp. The outcomes demonstrated that the ice cream developed had better texture and emulsification capabilities and a moderate level of fat [77]. Also, in the category of dairy products, Gavril (Rațu) et al. [78] incorporated pumpkin peel powder into yogurt and resulted in enhancements in the nutritional composition, namely in terms of β-carotene and bioactive substances. The sensory evaluation results indicated that the addition of pumpkin peel did not adversely affect the overall acceptance of the yogurt. In fact, some samples (YPP2) demonstrated preferred sensory characteristics over the control. Therefore, the study suggests that incorporating pumpkin by-products like pumpkin peel can contribute to sustainable food production and provide consumers with more diverse food choices that offer enhanced nutritional and sensory attributes.

Another study was also conducted to look into the effects of adding pumpkin seed powder to cereal milk that had already been fermented with *Lactobacilli* spp. and *Bifidobacteria* spp. cultures for the production of non-dairy probiotic goods [80]. The outcomes showed that adding pumpkin seed powder to cereal milk enhanced its physicochemical and sensory qualities.

Interest in researching and producing blended juices has grown since they reflect high-quality consistency and increased nutritional or phytochemical qualities. A study was conducted to develop a juice fortified with vitamins, antioxidants, and minerals derived from pumpkin pulp. The results of the study revealed that adding mango and strawberry juice to pumpkin juice improved the sensory quality [76]. 

#### Additional Applications of Pumpkin By-Products

Pumpkin seeds and pumpkin peels are very capable of being transformed and used to produce valuable goods with higher value, such as edible or biodegradable film. In order to create biodegradable films, defatted pumpkin seed meal, and peels were effectively used, and bio-based films made with co-products helped to lessen the environmental problem associated with composting. Such a film is suitable for baked goods, cakes, and desserts. These wastes have a high carbon content and could be desirable renewable substrates for producing added-value products. Cell viability was examined in wastes made from pumpkin peel and pulp (30:70 *w/w*), which had been treated with *Lactobacillus casei* at an initial inoculum size of 9 log CFU/mL and incubated at 37 °C. Increased cell viability after 24 h of fermentation showed that pumpkin was an effective carrier substrate for probiotics and an adequate carbon supply for their fermentation [83]. In their study, Lalnunthari et al. [51] investigated the production of an edible film by utilizing pectin and protein derived from pumpkin seeds and peels, respectively. Edible film, a type of coating material, is utilized as packaging or wrapping to protect products and culinary ingredients. They can be made by forming a film with proteins, polysaccharides, pectin, and lipids. The authors discovered that the procedure of various variables had an impact on the extraction of protein and pectin. Lalnunthari et al. [51] discovered a 69.89 ± 2.90% pectin production from pumpkin peels when the optimal conditions were an extraction temperature of 89.98 °C, an extraction time of 13 min, and a pH of 2.85. The films showed good elongation (9.74 ± 0.46%) and the greatest tensile strength values (1401 ± 5.4 kPa). Such edible film may be employed in the production of snack-friendly foods such as chopped fruit, baked goods, confectionery, etc. Based on minimally processed pumpkin residue extract (0 to 6%), glycerol, cassava starch, and oregano essential oil (0 to 2%), research by Dos Santos Caetano et al. [84] generated biodegradable films. The addition of pumpkin residue is essential since it gives the film opacity.

The manufacture of bioethanol from pumpkin peel wastes demonstrated the potential benefits of these materials. Artificial neural networks (ANNs) with estimation and prediction capabilities were employed in the study to optimize the bioethanol production process from waste pumpkin peel. This study utilized a multilayer feed-forward neural network that was developed using an error back-propagation learning approach. The mechanism involves hydrolyzing the reducing sugars present in the pumpkin peel using the enzyme amyloglucosidase, which breaks down the polysaccharides into fermentable sugars. Subsequently, these sugars undergo fermentation by the yeast *Saccharomyces cerevisiae*, resulting in the production of 50.60 g/L of bioethanol. This optimized process, guided by ANN predictions, effectively enhances the yield of bioethanol from pumpkin peel waste [85]. Furthermore, it was shown that the pumpkin peel contains a significant amount of carbohydrates, specifically 65.30%, along with fiber, water, protein, ash, and a tiny amount of fat. According to this information, pumpkin peel may be an efficient source of glucose. The ideal parameters for the fermentation process were anticipated based on time, temperature, substrate loading, α-amylase, and amyloglucosidase concentration. The results showed that the key factors affecting the levels of reducing sugar and bioethanol were the amount of substrate used and the temperature during fermentation.

H_3_PO_4_ was employed as a chemical agent to create activated carbon using pumpkin seed shells [86]. The structure of the pores of the activated carbon was dramatically impacted by carefully adjusting the impregnation proportion and activation temperature. So, pumpkin seed shells could serve as a different raw material for producing commercially viable activated carbon.

## 5. Various Techniques for the Extraction of Bioactives from Pumpkin By-Products

Extraction is the most important step, which is normally carried out with various solvents, acids, alkalis, and hydrodistillation methods depending on the type of extracted component. 

### 5.1. Conventional Techniques

Conventional extraction (CE) is among the most popular techniques. The solubility and volatility of the compounds in the chosen solvent have an impact on extraction efficacy. While phenolic chemicals are easily soluble in polar protic media (methanol, ethanol, acetone), carotenoids are fat-soluble in polar aprotic solvents (acetone, ethanol, acid acetic), or nonpolar solvents (ethyl acetate and benzene). Natural pigment extraction traditionally involves employing Soxhlet extraction techniques using organic or inorganic solvents, maceration, or hydro-distillation. Water or diluted alcohol is typically used for the conventional extraction of pigments that are water soluble, while non-polar solvents are used for the extraction of pigments that are lipophilic (hexane, acetone, methanol). The majority of them are harmful in nature, but because they are volatile, they can effectively break down target pigments to make removal easier. Instead of being technically advantageous, these solvents can pollute the environment and are dangerous for human consumption [87]. 

Conventional extraction methods have poor extraction efficiency, high energy consumption, take a long time to extract, and are not economically profitable. Due to these issues, innovative ecologically friendly methods are required to enable efficient extraction using green solvents derived from renewable resources, such as plant-based edible oils.

The effects of various extraction methods for carotenoids in pumpkin pulp were examined in a study. Temperature (15, 30, and 45 °C), extraction period (8, 12, and 16 h), and the solid-to-solvent ratio (1:50, 1:100, and 1:150) were all optimized for carotenoid extraction. To purify nonpolar carotenoids from pumpkin pulp, nonpolar solvents like lycopene and β-carotene are necessary [88]. Moreover, it was demonstrated that enhancing the solid-to-solvent ratio to a maximum of 1:150 improved carotenoid production.

### 5.2. Modern Extraction Techniques

New techniques have been introduced as a result of the limitations of conventional procedures. Traditional extraction techniques are characterized by the difficulties of obtaining high purity, the use of costly solvents, longer extraction durations, the potential for heat-labile chemical degradation, and low extraction selectivity. There are currently various novel and developing methods being employed for the extraction process.

Due to many advantages, including the need for minimal solvents, speed, convenience, and the ability to boost extraction yield while protecting pigments from deterioration and improving the quality of natural colorants, these approaches are more popular than traditional extraction methods. An overview of the extraction procedures for the valorization of pumpkin by-products is shown in Table 2.

#### 5.2.1. Ultrasound-Assisted Extraction (UAE)

UAE employs mechanical vibrations with an ultrasonic frequency greater than 20 kHz. This causes a cavitation effect, which speeds up the mass and heat transmission in the particles, disrupting cells, and enabling chemical release. Using ultrasound in the extraction process enables improved preservation of the bioactives’ properties. This technique significantly enhances extraction efficiency, reduces the necessary temperature, and shortens the extraction time [96].

Song et al. [90] evaluated the efficacy of ultrasound-assisted extraction (UAE) technology compared to conventional solvent extraction. Due to the UAE technique’s great efficiency, quick extraction time, and straightforward and uncomplicated operation, it is generally known that it can be employed to help extract carotenoids from natural sources. The study investigated the impact of ultrasonic power and frequency, solvent type, time, and solvent-to-material ratio on trans-lutein production and total trans-carotenoids. The utilization of ultrasound-assisted extraction (UAE) resulted in a substantial quantity of trans-lutein and trans-carotenoids, exceeding the conventional solvent extraction method. This is due to the UAE’s ability to prevent degradation and isomerization of the compounds. Also, in the study using pumpkin peel, Song et al. [90] enhanced the UAE by using traditional methods to determine the optimal solvent for extracting carotenoids. They discovered that a blend of ethanol and petroleum ether was the most effective solvent. In order to achieve this purpose, researchers assessed the impact of the key parameters that affect the yield of carotenoids, including ultrasonic power, extraction duration, and liquid–solid ratio. UAE achieved a carotenoid content of 363 µg/g under optimal conditions (203 W, 30 min, and a solvent-to-material ratio of 31 mL/g), resulting in a 92% yield increase compared to conventional extraction methods.

Sharma and Bhat [97] used maize oil as an eco-friendly solvent and three distinct extraction techniques (ultrasound-assisted extraction, microwave-assisted extraction, and conventional extraction) to isolate carotenoids from the pulp and peel of two pumpkin cultivars. The carotenoid concentration in the samples obtained by UAE was higher compared to the samples obtained through MAE and CE using organic solvents (hexane and isopropyl alcohol). Additionally, it was discovered that the amount of carotenoids varied based on the plant portion, with the peel powder having a higher amount than the pulp powder. In comparison to traditional extraction (19.21 ± 4.39; 16.21 ± 2.52 µg/g), the results showed that total carotenoids were almost two times higher when using ultrasound (38.03 ± 4.21; 33.78 ± 1.76 µg/g). 

Gavril et al. [78] study aims to determine the optimal extraction parameters for an ultrasonic-assisted extraction technique to extract whole carotenoids and evaluate the antioxidant activity of pumpkin peel. The most effective parameters for extracting carotenoids from pumpkin peel were a temperature of 80 °C, a solvent ratio of 10 mL, and an extraction period of 100 min. This resulted in a carotenoid concentration of 0.97 mg/g D.W. and an antioxidant activity of 7.25 µM TE/g D.W.

#### 5.2.2. Microwave-Assisted Extraction (MAE)

MAE is a method that combines conventional solvent extraction with nonionizing electromagnetic waves at microwave wavelengths between 0.3 and 300 GHz. By heating the entire sample simultaneously, the compounds are subsequently extracted because of the effects of dipole rotation and ionic conduction. The technique makes use of the particle-by-particle absorption of radiation by the material. The sample is heated and transfers the heat to the extractant as a result of employing a solvent that is not polar and does not absorb microwave radiation. The method is cost-effective since it shortens the extraction process and uses less solvent. Dipole particles move during microwave heating and generate heat energy via friction. The factors that can affect MAE efficiency include power, frequency, extraction duration, temperature, matrix moisture, solvent type, and the ratio of solid to liquid [98,99].

Hexane and ethanol were used as the extraction solvents in Ferreira et al.’s [93] comparison of UAE, MAE, and the traditional method for recovering phenolic components from pumpkin seeds (*Curcubita* sp.); the highest phenolic content and antioxidant activity were acquired using MAE under subcritical solvents (ethanol and water, 8.12 and 8.71 mg GAE/g, respectively) with good extraction yields [93]. However, the extract produced with subcritical ethanol had a higher concentration of phenols, yielding 83.95 mg GAE/g of extract as opposed to 55.52 mg from subcritical water. These results, 3.93 mg GAE/g matrix and 42.42 mg GAE/g extract, were better than those from traditional extraction. The authors explained that this is caused by the decrease in solvent polarity that occurs in subcritical conditions, which encourages the solubilization of a variety of phenolic compounds.

#### 5.2.3. Supercritical Fluid Extraction (SFE)

SFE operates at higher pressures and temperatures than the critical points of fluids such as CO_2_, water, methanol, ethanol, n-butane, and ethylene. The most often utilized supercritical fluid is CO_2_, which is readily available in high purity, affordable, flammable, chemically inert, and recyclable. Low temperature and low pressure, crucial for extracting natural compounds, especially those with thermally unstable components, are the key benefits of employing supercritical CO_2_. The critical point for CO_2_ is at 31.1 °C (304.2 K) and 7.3 MPa (72.8 bar), allowing for work at ambient temperature and low pressure, which is suitable for the extraction of thermolabile substances [100]. 

By extracting pumpkin seed oil and the fruit’s peel, a high-quality extract that is acceptable for use in food, medicine, and cosmetics has been produced [100]. By including the pumpkin peel during the oil extraction process, bioactive components from the peel, including carotenoids, phytosterols, tocopherols, and antioxidant activity, were added to the extract. The solvent solution for the simultaneous extraction was sub- and supercritical CO_2_. The utilization of optimal pressured extraction conditions, including temperature and pressure, using CO_2_ resulted in the production of a higher quality extract compared to those obtained through conventional and ultrasonic methods, as evidenced by the concentration of β-carotene, phytosterols, tocopherols, phenolics, antioxidant activity, and thermal stability. 

Mitra et al. [101] examined the Soxhlet method of extracting oil from pumpkin seeds using hexane and CO_2_ supercritical extraction, achieving comparable yields with both methods. SFE also made it possible to shorten extraction times and use less organic solvent in both studies. Consequently, the oil yield obtained at the ideal conditions (68.1 °C, 94.6 min, 32,140 kPa) was 31%. Supercritical CO_2_ extraction was also used by Wang et al. [61] to extract β-carotene (18.50 mg/100 g sample) from pumpkin seeds.

#### 5.2.4. Deep Eutectic Solvents Extraction (DESE)

Deep eutectic solvents are new green solvents that recently have been discovered that are nontoxic, affordable, biodegradable, and safe for the environment; they are a mixture of two or three components generating hydrogen bonds. An organic salt (quaternary ammonium or phosphonium salt) serves as the hydrogen bond acceptor, whereas alcohol, amino acids, sugar, organic acids, etc., serve as the hydrogen bond donors [102]. Because they have the potential for recycling and make it simple to purify the extracted bioactive components, these solvents are seen as intriguing alternatives. To extract β-carotene from refuse pumpkin materials, Stupar et al. [95] utilized hydrophobic natural deep eutectic solvents derived from fatty acids. One of the most effective natural deep eutectic solvents was selected as caprylic acid:capric acid (3:1), yielding 151.41 µg/mL of β-carotene at the ideal conditions of 50 °C, 60% (52.5 W/cm^3^) ultrasonic power, and a solvent-to-solid ratio of 7 mL/g over the course of 10 min of extraction. In order to develop a sustainable and effective extraction method of pigment from pumpkin peels, Huang et al. [103] conducted an experiment using different combinations of deep eutectic solvent alcohol and two aqueous phase systems. Subsequently, they implemented a Box–Behnken design and ultrasound-assisted cellulase to optimize the parameters. The results showed that the extraction solvents utilized were DES (choline chloride:triethylene glycol, 1:3) and ethanol (6:4). At a solid–liquid ratio of 1:40 g/mL, a cellulase dose of 2.10%, and an ultrasonic power of 300 W for 40 min; the pigment yield was 2.460 ± 0.037%.

## 6. Potential Health Benefits of Pumpkin By-Products

The therapeutic potential of pumpkin and its by-products against a variety of diseases is due to bioactives that are able to produce a distinct physiological effect in the human body. Therefore, bioactive substances are of great relevance for human health after evaluating studies on the chemical, biological, and pharmacological features of pumpkin and taking its by-products, taking peels, seeds, and fruit into consideration.

### 6.1. Antioxidant Activity

Antioxidants are useful in managing and preventing various diseases by preventing the accumulation of free radicals. Pumpkin by-products are abundant sources of various antioxidant substances such as flavanoids, tocopherols, phenolic acids, and carotenoids that preserve proper oxidative stability [99]. By using acidified methanol to extract pumpkin seeds, Yasir et al. [104] found that the extraction yield increased by 1.4 to ten times, the phenolic content increased (149.5–396.4 mg GAE/g), the DPPH radical scavenging activity increased, and the geno-protective activity was enhanced using the pBR322 plasmid assay.

In order to assess the antioxidant properties of pumpkin seeds and shells, Saavedra et al. [2] implemented numerous extraction solvents and dehydration techniques. Applying a 70% ethanol solution to both the seeds and shell samples showed that the presence of 2,2-diphenyl-1-picrylhydrazyl (DPPH) free radicals was reduced by 18.92–70.96%. The way in which various dehydration techniques suppress DPPH radicals is significantly different. The freeze-dried samples exhibited inhibition ranging from 1.47% to 52.41%, whereas the oven-dried samples at 65 °C showed inhibition ranging from 2.65% to 72.36%. The scavenging activity of pumpkin peel and pulp extract is around 80% at a concentration of 50 mg/mL [36].

### 6.2. Antihypertensive and Cardioprotective Activity

Blood pressure in the arteries is constantly high due to the long-term health condition known as hypertension, which is also a risk factor for cardiovascular illnesses. Several medicinal components, including mono- and polyunsaturated fatty acids like omega 3, omega 6, oleic acid, and linoleic acid, are abundant in pumpkin seed oil and play a significant role in lowering the risk of hypertension and cardiovascular illness [105]. In mice with high cholesterol, a supplement of pumpkin seeds showed anti-atherogenic and hepatoprotective effects [106].

Zuhair et al. [107] reported that pumpkin seeds exhibit hypotensive effects. Pumpkin seed oil demonstrated a favorable drug interaction outcome when administered in animal models with commonly used hypotensive medicines such as felodipine.

### 6.3. Anticancer Activity

The phytoestrogenic chemicals found in pumpkin seeds make them a promising applicant for avoiding hormone-dependent cancer. The anticancer activity of the seeds extract, for instance, was tested on MCF7, Jeg3, and BeWo (chorionic carcinoma), and the results showed good cytotoxic activity (10 or 50 µg/mL) and significant estradiol production. These findings indicated that, like many phytoestrogenic substances, pumpkin seeds, which are high in lignans and flavones, exhibit a biphasic effect through a variety of pathways with both estrogenic and antiestrogenic activities [108]. The seed oil of *C. pepo* effectively inhibited the MCF7 (breast cancer) cell line with an IC50 of 0.4 mg [109].

In a study comparing the cytotoxic effects of various pumpkin portions on the HEPG2 (liver carcinoma) and MCF7 cell lines, all extracts (pulp, peel, seed, and defatted seed extracts) showed strong cytotoxic effects with IC50 values ranging between 0.60 and 5.03 g/mL and 0.40–1.01 µg/mL, respectively [38]. Pectin has been identified as the most favorable polysaccharide found in pumpkin peels or pulp. Numerous papers have described pectin as a bioactive substance that effectively prevents cancer [110]. The ethanol-based extracts of *C. maxima* seed demonstrated complete suppression of esophageal adenoid cystic carcinoma cell lines at a concentration of 100 µg/mL, indicating strong anticancer activity [111].

Numerous carotenoid pigments, which have been demonstrated to lower the risk of cancer, are abundant in pumpkin seed oil. It has been claimed that pumpkin seed consumption has an adverse relationship with the development of several cancers, including lung, rectal, and breast cancer [66].

It is claimed that consuming 20–40 mg/kg of pumpkin seed oil orally for 20 days can be beneficial in reducing the enlargement of the prostate gland caused by testosterone [112]. Studies conducted in both living animals (in vivo) and laboratory settings (in vitro) have shown that pumpkin inhibits prostate cancer. Furthermore, a study on Sprague Dawley rats showed that applying pumpkin seed oil successfully decreased the progression of testosterone-induced hyperplasia. These findings indicate that pumpkin seed oil may have substantial advantages in the management of benign prostatic hyperplasia [113].

### 6.4. Anti-Hyperlipidemic Effect

In their study, Majid et al. [114] examined the effects of pumpkin seed oil on human subjects under controlled conditions. The findings demonstrated a significant decrease in endpoint LDL (low-density lipoprotein) cholesterol and a substantial increase in HDL (high-density lipoprotein) cholesterol. The experimental findings suggest that pumpkin seed oil exhibits hypolipidemic and antihypertensive effects since it reduces LDL levels and elevates HDL levels. 

Pumpkin seed powder contains arginine, a precursor of nitric oxide, at a concentration of 2.6%. Arginine is associated with apoptosis, blood pressure regulation, cardiac function, and inflammatory response [55]. A study examined the impact of pumpkin seed extract, which consists mainly of arginine, on rats with hyperlipidemia. The study discovered that administering pumpkin seed extract to these rats resulted in a rise in nitric oxide synthesis, most likely attributed to arginine in the extract. In addition, the production of nitric oxide leads to a decrease in the expression of the vascular cell adhesion molecule, hence reducing the oxidation of LDL [115]. Thus, it can be concluded that altering lifestyle and incorporating regular consumption of pumpkins into a diet can be a helpful dietary strategy to treat hypercholesterolemia.

### 6.5. Anti-Diabetic Effect

Studies have indicated that dietary fibers, like pectin found in pumpkin skins, can inhibit the digestion of starch. This can be beneficial in managing diet-related conditions such as diabetes [116]. Adams et al. [117] identified several bioactive components in pumpkin that contribute to the reduction in blood glucose levels. Specifically, pectin and non-pectin polysaccharides derived from the epidermis and pulp, as well as hypoglycemic proteins and seed oils from pumpkin seeds, were found to be effective in lowering blood glucose. In studies involving alloxan-induced diabetic rats, protein-bound polysaccharides were reported to significantly reduce blood glucose levels, increase plasma insulin concentrations, and enhance glucose tolerance. In rats fed a diet containing 2% cholesterol, a combination of pumpkin seeds with flax seeds or purslane seeds was shown to have an anti-atherogenic impact by lowering total cholesterol, triglycerides, and total lipids in both the liver and serum [118]. By-products from pumpkins are a plentiful source of several nutraceuticals that can treat hypercholesterolemia and diabetes. For instance, the high dietary fiber content of pumpkin peels may help avoid hypercholesteremia and diabetes [115]. The anti-diabetic properties of *C. maxima* seed are attributed to the inhibitory impact of its aqueous extract on α-amylase and α-glucosidase enzymes. The anti-diabetic effectiveness of a newly discovered protein-bound polysaccharide from pumpkin seeds was assessed based on its ability to inhibit α-amylase. When administered at a concentration of 5.0 mg/mL, it was found to exhibit α-amylase inhibition with a percentage of 41.3 ± 2.1%. Pumpkin-based polysaccharide is a useful treatment for diabetic individuals [119].

### 6.6. Anti-Inflammatory Effect

The management of many diseases can be aided by preventing inflammation, which is a risk factor for many pathological disorders like diabetes, cancer, and other concerns. The anti-inflammatory effects of pumpkin by-products are attributed to their rich content of polyphenols and other antioxidant compounds, which help in reducing inflammation by neutralizing free radicals and modulating inflammatory pathways. In several disorders, it has been claimed that pumpkin seed oil can reduce and avoid inflammation. In rats subjected to pyloric ligation, water immersion stress, and indomethacin-induced ulcer procedures, a newly obtained tetracyclic triterpenoid from pumpkin seeds oil at a dose of 300 µg/mL showed optimal antiulcer action, with the percentage of inhibition being 55.7%, 67.1%, and 59.1%, respectively [120]. Through a decrease in DNA fragmentation, Bardaa et al. [121] demonstrated that oral therapy of azathioprine-induced cutaneous lesions in rats with pumpkin seed oil (4 mL/kg b.wt) improved macroscopic, morphometric, and histological results compared to the untreated group. The isolation of 3-hydroxycholest-7-en-24-one from *C. pepo* seeds activated macrophages by inhibiting the generation of nitric oxide in RAW 264 cells [122]. 

### 6.7. Antimicrobial Effect

The linoleic and oleic acids in the seed oil may also have strong antibacterial properties against *S. aureus*, with a zone of inhibition of 15 mm, according to Adnan et al. [109]. In tests against *Bacillus subtilis*, *S. aureus,* and *E. coli,* pumpkin seed extracts in petroleum ether and methanol at concentrations of 10, 50, 100, 200, 500, and 1000 g/mL were successful [109].

The disc diffusion method reveals that the methanolic and ethanolic extracts derived from pumpkin peel exhibit antibacterial properties, resulting in an inhibitory zone ranging from 6 to 10 mm [123]. According to Ahmad and Khan [124], research has shown that pumpkin seed oil has strong antibacterial action against *Pseudomonas aeruginosa*, *Candida albicans*, *Acinetobacter baumanii*, *Enterococcus faecalis*, *Klebsiella pneumonia*, *E. coli*, and *Staphylococcus aureus*.

Asif et al. [125] found that pumpkin peel extract showed significant antibacterial properties against four bacterial strains. This was determined using the disc diffusion method, and the inhibition zone values ranged from 10 to 15 mm. The high antioxidant and antibacterial capacity of pumpkin peel suggests that this section of the fruit could be used to develop antioxidant-rich functional foods.

A polysaccharide derived from pumpkin seeds was shown to have antibacterial properties. At a dosage of 0.5 mg/mL, significant suppression was seen against the bacteria *E. coli* (12.8 ± 1.0 mm) and *B. subtilis* (7.2 ± 0.3 mm), whereas a modest zone of inhibition was seen against *Pichia fermentans* (2.3± 0.2 mm) and *Staphylococcus aureus* (2.3 ± 0.2 mm) [3,61]. 

### 6.8. Other Health Benefits

Rezig et al. [126] showed that pumpkin seed oil decreases the pressure inside the urethra and bladder, by lowering crystal growth, which lowers the chance of bladder stones, among other disorders. According to Baek et al. [127], the greater iron concentration in pumpkin seeds aids in alleviating the anemia brought on by iron scarcity. Consuming raw and processed pumpkin seeds may be beneficial for treating depression. Trypsin might be inhibited and the Hageman factors could be activated by protein isolates from pumpkin seeds. Additionally, pumpkin seeds are used in a variety of cosmetic products, including dry facial masks, body scrubbers, body butter, and scrubs for the body [13]. A study was conducted to see how aspartame and pumpkin seed oil interacted. Reports suggest that administering a dosage of 4 mL/kg of pumpkin seed oil mixed with water for a duration of four weeks can mitigate the adverse effects of aspartame and provide protection to the liver. This treatment with pumpkin seed oil effectively preserved the globulin levels, liver enzymes (aspartate transaminase, alkaline phosphatase), albumin, and bilirubin [128].

By preserving the gastric mucosa in a dose-dependent manner, pumpkin seeds have additionally demonstrated anti-ulcerative capabilities [120]. Vitamin C-rich *C. pepo* pulp is used as a remedy for enteritis, dyspepsia, and stomach problems. Sarkar et al. [129] found that albino rats given pumpkin fruit pulp extract showed higher alkaline phosphate activity, thinner mucosal lining, and lower ulcer index. In contrast, albino rats given aspirin had reduced alkaline phosphate activity, thicker mucosal lining, and higher ulcer index. This indicates that the pulp of *C. pepo* has properties that protect the stomach and duodenum from damage and can be used to treat ulcers.

Researchers’ attention is always on developing more efficient and affordable methods of wound healing since they believe that current medical treatments are insufficient [130]. Pumpkin seed oil’s high concentrations of plant sterols, tocopherols, and polyunsaturated fatty acids have a beneficial impact on wound healing [121]. It has also been noted that similar findings regarding pumpkin peel exist [22].

## 7. A Biorefinery Approach for the Valorization of Industrial Pumpkin By-Products

Several processing options for their integrated valorization might be addressed, taking into consideration the composition of the pumpkin by-products and the large range of high-added-value bio-products available. To do this, biorefineries should be developed that concentrate on the selective separation of their primary constituents and their afterward transformation into building block chemicals and their derivatives. In order to extract fatty acids and/or oils from the pumpkin by-products, a traditional extraction using organic solvents could be chosen as the initial step. Based on the Alonso et al. [131] and Gullón et al. [132] studies, the solid produced after this initial processing might be submitted to an autohydrolysis procedure to extract the key components. Two distinct fractions would result from this processing: a liquid fraction rich in oligosaccharides and phenolic chemicals, and a solid fraction high in cellulose and lignin. To recover oligosaccharides and other molecules with antioxidant activity individually, further ethyl acetate extraction could be performed on the resulting liquid phase. A delignification process (using alkali or deep eutectic solvents) on the autohydrolyzed solid enables the solubilization of lignin, producing a solid residue primarily made of cellulose. The dark fluid would then be acidified to precipitate lignin. The depolymerization of lignin for the generation of aromatic building blocks with significant application potential might also be discussed as a means to develop a practical biorefinery. Several techniques, including base-catalyzed hydrolysis, pyrolysis, hydrogenolysis, or oxidation, could be used to carry it out. Following the delignification process, the resulting solid can be utilized for either the enzymatic hydrolysis of cellulose to produce saccharification or the acid hydrolysis to generate nanocrystalline cellulose. The upgrading of the resultant glucose to various platform chemicals (such as bio-ethanol and succinic acids) through the fermentative process or its catalytic oxidation to furan derivatives are two intriguing ways that could be taken into consideration. Applying the principles of the circular economy, the biorefinery concept would enable the pumpkin by-products’ re-incorporation into productive processes as abundant and affordable raw materials, satisfying the needs of regulations addressing the prevention and management of waste [132,133,134,135].

## 8. Conclusions and Future Perspectives

Pumpkin and its by-products have drawn attention for its nutrient profile, pharmacological properties, and industrial usage, which may be related to its enormous number of phytochemicals and bioactive compounds.

Bioactives found in various pumpkin parts have been shown to lower the risk of certain chronic diseases. Due to its phytochemical profile, health benefits, and industrial uses, it is imperative to investigate the significance of pumpkins for consumers. A circular and sustainable economy centered on the pumpkin industry would be supported by the reuse of these biomolecules, which have applications in the food, pharmaceutical, and cosmetic industries. With the aim of eliminating food waste and lowering losses in manufacturing and supply chains, this strategy is pertinent to achieving sustainable development goals. In addition to having a higher cost–benefit ratio, eliminating food loss, increasing energy efficiency, and lowering environmental pollution, the bioactive compounds recovered using green extraction processes have considerable economic potential [136].

More research is required to determine the nutritional profile and potential industrial uses of pumpkins.

Pumpkin seeds are valued for their oil, rich in essential fatty acids, and as a strong source of proteins with essential amino acids. Extracted compounds need further clinical testing. Promoting pumpkin consumption in developing nations can reduce costs and offer therapeutic benefits.

Different foods may prevent oxidation using the phytochemical-rich bioactive substances in pumpkin and its by-products. Current processing techniques could be applied to demonstrate the functional influence that pumpkin by-products have on various food products. In addition, research can be carried out on the household consumption of pumpkin by-products.

The previously mentioned investigations indicate that pumpkin wastes, including peel and seeds, possess substantial potential to be employed as raw materials for various purposes. However, in order to maximize the production of the desired chemical, such as cellulose, pectin, bioethanol, etc., they require an appropriate pretreatment procedure. Future research must focus on improving the pretreatment process to extract the important lignocellulosic material from pumpkin wastes in its entirety.

Researchers, food industry specialists, and consumers can encourage healthy and sustainable nutrition by acknowledging and utilizing the potential of these by-products, while also presenting new and nutritious food options.

Consequently, every part of the pumpkin plant can be utilized in extracting phytochemicals that provide beneficial biological impacts on human health. By adopting this approach, the byproduct generated from pumpkin cultivation and processing might be effectively repurposed. These items could potentially be repurposed and altered for use in new commercial goods, thus supporting a circular economy framework that is recognized for its economic and ecological benefits. 

Future research should focus on optimizing extraction methods, the economic feasibility of using pumpkin waste, exploring new applications for these by-products, and integrating them into food products to enhance nutritional value and sustainability in the food industry. Integrating pumpkin by-products into food industry products and processes can offer several benefits: nutritional value, innovation in product development, cost efficiency, support for circular economy, and sustainability.

## Figures and Tables

**Table 1:** Potential food utilization of different forms of pumpkin by-products.

Form of the Pumpkin By-Product	Food Product	Functional/ Technological Benefits	References
Pumpkin peel flour	Bread	The bread with 5% pumpkin peel flour showed good characteristics with high protein content, raw fiber, and a lesser amount of carbohydrate.	[11]
Pumpkin seed flour	Muffins	Compared with other muffins, muffins containing 33% seed flour had a better sensory profile and improved nutritional value.	[69]
Pumpkin peel flour	Biscuits	Biscuits prepared with 20% pumpkin peel flour were most appreciated with good taste and appearance.	[67]
Pumpkin pulp flour	Gluten-free pasta	The gluten-free pasta with 25% (13.5 g/100 g) pumpkin flour had the most desirable overall acceptability and sensory attributes among all formulated pasta. The moisture content, ash, cooking yield, and a* were increased by the partial replacement of the corn flour with pumpkin flour.	[70]
Dried pumpkin powder	Beef sausage	The sample formulated with 30% dried pumpkin powder recorded the lowest moisture content (64.22%) compared with (72.43%) for the control sample. Significant increase (*p* < 0.05) in fat (9.30%) and protein (20.55%) content was also recorded.	[72]
Pumpkin seed	Chicken burgers	Improved stability during the period of storage.	[73]
Pumpkin seed kernel flour	Beef meat balls	Fat replacement.	[74]
Pumpkin seed and pulp	Beef patties	There was no change in the texture and a decrease in the moisture content.	[75]
Pumpkin pulp	Pumpkin-based juice blends	The results reveal that the pumpkin juice developed in this study had high levels of hydration, crude protein, fiber, ash, and carbohydrates, suggesting that it is a rich source of these essential nutrients. The sensory analysis indicated that consumer groups deemed the pumpkin-based juice blends acceptable.	[76]
Pumpkin pulp	Yogurt	Increase health benefits.	[77]
Pumpkin peel	Yogurt	Adding pumpkin peel powder to yogurt enhanced the nutritional profile, namely in terms of β-carotene and bioactive components. The use of powder also beneficially impacted the yogurt’s textural characteristics, demonstrating improved consistency and mouthfeel.	[78]
Pumpkin seed	Ice cream	Augmenting the protein content boosts the level of fullness and improves sensory attributes.	[79]
Pumpkin seed powder	Cereal milk	Incorporating pumpkin seed powder improved the sensory and physicochemical properties of cereal milk. The refrigerated storage extended the shelf life of cereal milk to 9 days.	[80]
Pumpkin pomace powder	Cheese	The addition of PP powder to the cheeses led to an increase in both the carotenoid content and antioxidant activity, resulting in improved sensory evaluation scores.	[81]

**Table 2:** Overview of extraction processes for pumpkin by-products.

Pumpkin By-Products	Process Conditions	Compounds	Yield	References
Pumpkin peels	CE Methanol 80% 1:33 *w/v*	Phenolic compounds	319–529 mg GAE/100 g D.W.	[12]
Pumpkin peels	CE 70 °C/0.5 h Water/70% ethanol/70% methanol 1:25 *w/v*	Phenolic compounds	200–1069 mg GAE/100 g D.W.	[2]
Pumpkin peels	CE, on ice until colorless THF:methanol (1:1 *v/v*) containing 0.1% BHT, 1:1.5 *w/v*	Carotenoids	40–130 mg/kg F.W.	[9]
Pumpkin peels	CE, until colorless acetone:hexane (1:1 *v/v*)	Carotenoids	12–1751 mg/kg D.W.	[89]
Pumpkin peels	UAE, hexane:acetone (3:1 *v/v*) Central composite design extraction temperature (6.25–98.75 °C), extraction duration (13.98–128.98 min), and solvent ratio (0.23–50.23 mL)	Carotenoids and antioxidant activity	0.53 to 1.06 mg/g D.W. and 0.34 to 7.28 µM TE/g D.W.	[78]
Pumpkin peels	UAE 20 °C/0.5–0.83 h, Ethanol:petroleum ether 1:30–1:40 *w/v*	Carotenoids	98–239 μg trans-lutein/g D.W.	[90]
Pumpkin peels	UAE 25 °C/30 min corn oil as an alternative solvent, amplitude-20%,	Carotenoids and phenolic compounds	38.03 ± 4.21 µg/g of Oil Extracts 588.68 ± 7.26 mg GAE/g of Extract	[3]
Pumpkin peel extract	Subcritical water extraction (SWE) and SFE, SWE-Water, and SFE–SC–CO_2_ 120 °C/3 h/5 MPa (SWE) 60 °C/3 h/25 MPa (SFE)	Carotenoids	15.22 mg/100 g extract (SWE) and 11.48 mg/100 g extract (SFE)	[91]
Pumpkin peels	SFE 59 °C/0.5 h CO_2_, 15.5% Ethanol	Carotenoids	85% total carotenoid recovery	[92]
Pumpkin peels	CE, on ice until colorless Hexane containing 0.1% BHT, 1:1.5 *w/v*	Tocopherols	5–13 mg/kg F.W.	[9]
Pumpkin seeds	CE 70 °C/0.5 h Water/70% ethanol/70% methanol, 1:25 *w/v*	Phenolic compounds	95–343 mg GAE/100 g D.W.	[2]
Pumpkin seed	CE 20 °C/16 h 70% acetone/100% dichloromethane, 1:25 *w/v*	Phenolic compounds	132–612 mg GAE/g D.W.	[2]
Pumpkin seeds	CE Ethanol–hexane 80 °C, 4 h	Phenolic compounds	3.5–42.4 mg GAE/g D.W.	[93]
Pumpkin seeds	MAE, hexane, 5–30 min/200 W, 1:10 (g/mL), 20 kHz	Oil	51–62.5%	[94]
Pumpkin seeds	MAE Ethanol–water 100–150 °C, 20 min, 2.45 GHz	Phenolic compounds	35–84 mg GAE/g D.W.	[95]
Pumpkin seeds	UAE Ethanol–water 40 °C/20 min, 1:10 (g/mL), 100 W, 20 kHz	Phenolic compounds	34.2 mg GAE/g D.W.	[93]
Pumpkin seeds	CE 0.05 h 0.1% HCOOH in methanol 70% 1:10 *w/v*	Phenolic compounds	379 mg GAE/100 g D.W.	[47]
Pumpkin seeds	UAE, 0.25 h Ethanol 50%/Methanol 80%/Acetone 1:5 *w/v*/1:15 *w/v*	Phenolic compounds	34–113 mg GAE/ 100 g F.W.	[48]
Pumpkin seeds	CE on ice until colorless THF:methanol (1:1 *v/v*) containing 0.1% BHT, 1:1.5 *w/v*	Carotenoids	7–32 mg/kg F.W.	[9]
Pumpkin seeds	CE on ice, until colorless Hexane containing 0.1% BHT, 1:1.5 *w/v*	Tocopherols	49–93 mg/kg F.W.	[9]
Pumpkin seeds	UAE 0.17 h Dichloromethane:Methanol (1:4 *v/v*) 1:50 *w/v*	Tocopherols	29–71 mg/100 g D.W.	[14]
Pumpkin seeds	CE Chloroform:methanol (2:1 *v/v*) 1:20 *w/v*	Fatty acids/oil	440–524 g/kg F.W.	[9]
Pumpkin Peels	MAE 45 °C/130 W/30 min corn oil, 1:10 ratio	Carotenoids and phenolic compounds	34.94 ± 3.60 µg/g of Oil Extracts 554.54 ± 10.25 mg GAE/g	[3]
Pumpkin by-products	UAE, Caprylic acid:Capric acid (3:1) 50 °C,/52.5 W/cm^3^ ultrasonic power/7 mL/g ratio/10 min	β-carotene	15,141 mg/100 g	[95]

GAE, gallic acid equivalents; D.W., dry weight; BHT, 2,6-di-tert-butyl-4-methylphenol; PVP, polyvinyl-pyrolidone; RME, reverse micelle extraction; CTAB, cetyltrimethylammoniumbromide; ATPS, aqueous two-phase system; FPU, filter paper unit; SFE, supercritical fluid extraction; UAE, ultrasound-assisted extraction; CAE, chlorogenic acid equivalents; EAE, enzyme assisted extraction; F.W., fresh weight; MAE, microwave-assisted extraction; CE, conventional extraction; r.m. raw material; THF, tetrahydrofuran; PLE, pressurized liquid extraction.

## Data Availability

No new data were created or analyzed in this study. Data sharing is not applicable to this article.
